# Stereotactic navigation versus ultrasound guidance in placing IRE applicators in a liver phantom

**DOI:** 10.1038/s41598-021-00505-1

**Published:** 2021-10-26

**Authors:** David Stillström, Benjamin Eigl, Jacob Freedman

**Affiliations:** 1 Division of Surgery, Department of Clinical Sciences, Karolinska Institutet at Danderyd Hospital, Stockholm, Sweden; 2 Department of Surgery and Urology, Danderyd Hospital, 182 88 Stockholm, Sweden; 3 CASCINATION AG, Bern, Switzerland

**Keywords:** Health care, Medical research

## Abstract

The aim of this study was to compare the accuracy of stereotactic CT-guided navigation and ultrasound guided navigation for placing electrodes in Irreversible electroporation in a liver phantom. A liver phantom with multiple tumours was used and interventionists placed four IRE electrodes around each tumour guided either by stereotactic CT-guided navigation or ultrasound. The goal was to place them in a perfect 20 × 20 mm square with parallel electrodes. After each treatment, a CT-scan was performed. The accuracy in pairwise electrode distance, pairwise parallelism and time per tumour was analysed. Eight interventionists placed four electrodes around 55 tumours, 25 with ultrasound and 30 with stereotactic CT-guided navigation. 330 electrode pairs were analysed, 150 with ultrasound and 180 with stereotactic CT-navigation. The absolute median deviation from the optimal distance was 1.3 mm (range 0.0 to 11.3 mm) in the stereotactic CT-navigation group versus 7.1 mm (range 0.3 to 18.1 mm) in the Ultrasound group (p < 0.001). The mean angle between electrodes in each pair was 2.7 degrees (95% CI 2.4 to 3.1 degrees) in the stereotactic CT-navigation group and 5.5 degrees (95% CI 5.0 to 6.1 degrees) in the Ultrasound group (p < 0.001). The mean time for placing the electrodes was 15:11 min (95% CI 13:05 to 17:18 min) in the stereotactic CT-navigation group and 6:40 min (95% CI 5:28 to 7:52 min) in the Ultrasound group. The use of stereotactic CT-navigation in placing IRE-electrodes in a liver phantom is more accurate, but more time consuming, compared to ultrasound guidance.

## Introduction

Local ablative therapies are well established techniques in the treatment of both primary and metastatic liver tumours^1,2^. Radiofrequency ablation (RFA) and microwave ablation (MWA) are the most commonly used methods^3,4^. Both RFA and MWA use heat to induce coagulative necrosis in tumour tissue and surrounding margins. If the tumour is located too close to major hepatic vessels, bile ducts or other heat sensitive structures an alternative method must be used. This is the primary role for irreversible electroporation (IRE) in the treatment of hepatic tumours^1,3^.

The IRE treatment is not based on a thermal effect. Multiple electrodes are placed around the tumour and short pulses with direct current at high voltage are applied between each pair of electrodes. This leads to formation of nano-pores in the cells’ lipid bilayer, causing a disruption of the cells homeostasis, leading to apoptosis^1,5–7^. Treating tumours close to heat sensitive structures is therefore possible since rise in temperature is low in the field of treatment.

For IRE, multiple electrodes must be placed in parallel and with a specific interelectrode distance to create a homogeneous ablation volume. As the treated tumours are situated close to sensitive structures, often in the central part of the liver, the accuracy of the electrode placement is crucial. This is more challenging and time consuming than placing a single applicator with other ablative methods. The electrodes can be placed under ultrasound guidance, using ultrasound with fused computed tomography (CT) images or magnetic resonance imaging (MRI), using conventional CT puncture techniques or with stereotactic CT-guidance^8^. Ultrasound, with or without contrast enhancement, is widely used but has its limitations, especially for deep sited lesions. The procedure is also highly dependent on the interventionist’s experience^9^.

Previous studies have proven the safety and efficacy of stereotactic CT-guidance for placement of single applicators in RFA and MWA^10–13^.

IRE electrodes, with the NanoKnife (AngioDynamics, Latham, NY) system, are typically placed 20 mm apart and as parallel as possible with a length of exposed electrode between 15 to 20 mm. In ultrasound guided IRE one challenge is that the point where the electrode should be placed is off target, i.e., outside the tumour, compared to RFA and MWA where the target point is typically located in the centre of the tumour. Another challenge with ultrasound is the targeting of tumours located high up in the liver close to the lung since air effectively blocks the ultrasound waves.

To optimise the electrode placement different methods of computer assisted guidance have been developed and shown superior results compared to conventional CT-guided electrode placement^14–16^. Our group has previously presented data on safety, efficacy and accuracy of stereotactic CT guided navigation in IRE treatment^17,18^.

The aim of this phantom study is to compare stereotactic CT-guided navigation with ultrasound guided navigation regarding the placement accuracy of IRE electrodes around hepatic tumours.

## Materials and methods

To compare the accuracy between stereotactic CT-guided navigation and ultrasound guided navigation, IRE electrodes were placed around tumours in a liver phantom with guidance of either modality.

### The phantom:

The commercially available Triple modality 3D abdominal phantom, model 057A (CIRS Tissue Simulation & Phantom Technology, Norfolk, VA, USA) was used. It is designed for use with ultrasound, CT and MRI. The phantom’s internal structures include the liver, the portal vein, two partial kidneys, a partial lung, the abdominal aorta, the vena cava, a simulated spine and six ribs. The liver has six lesions of which five were used in this study. The liver, tumours and surrounding tissues are visible on ultrasound and CT.

**Figure 1:**
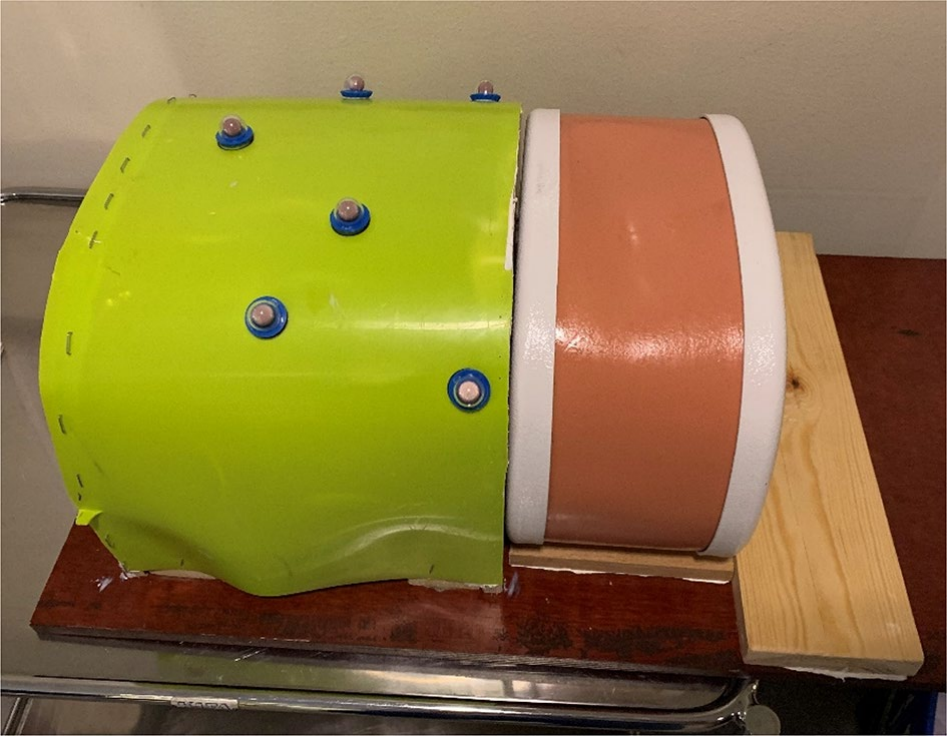
The phantom to the right with the simulated thorax to the left with the skin markers attached.

The phantom was extended with a simulated thorax where the single markers were attached (Fig. 1).

### Procedures

**Figure 2:**
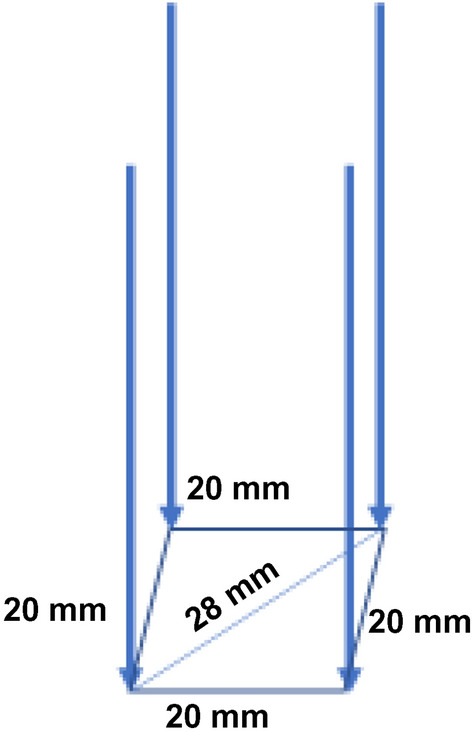
Geometry of four electrodes in a square.

In this study, electrodes from the NanoKnife system were used. For each tumour, the goal was to place four IRE-electrodes in a perfect 20 mm × 20 mm square with parallel electrodes at the same depth, with the centre of the tumour at 1 cm from the tip, regardless of the tumour size in the phantom (Fig. 2).

For each interventionist and group, the electrodes were placed in two sessions, three tumours in the first and two tumours in the second session.

For the CAS-One guided placements, an initial CT scan of the phantom was used for all planning and navigation. After each session, a control CT scan was acquired and uploaded to the system.

For the ultrasound-guided electrode placements, the electrodes were placed under live ultrasound monitoring and after each session a control CT scan was recorded and uploaded to the navigation system.

### Interventionists:

Five interventionists performed the ultrasound guided electrode placements, all with experience from interventional ultrasound and ablative treatments. Six interventionists performed the CAS-One guided electrode placements all with experience from CAS-One guided MWA and IRE. See Table 1.

**Table 1:** Interventionist: speciality, and previous experience.

Interventionist	US ablation	US biopsy	CAS ablation
1. Radiologist	> 100	> 1000	> 100
2. Radiologist	> 100	> 1000	> 100
3. Radiologist	> 100	> 1000	> 50
4. Radiologist	> 50	> 1000	
5. Radiologist	0	> 100	
6. Surgeon			> 100
7. Surgeon			> 100
8. Surgeon			> 100

### CAS-One

The CAS-One IR system (CAScination AG, Bern, Switzerland) is a user controlled, stereotactic accessory intended to assist in planning, navigation and manual advancement of one or more instruments, as well as in verification of instrument position and performance during Computed Tomography (CT) guided procedures. The system has previously been described^7,9,16,17^ For this study, six retroreflective markers were placed on the phantom thorax, which were detected by the optical camera and in the CT images to register the physical with the virtual space (see Fig. 2). For each tumour, a four-needle configuration with 20 mm interelectrode distance was planned on the multiplanar reconstruction of the CT image data. A tracked mechanical arm was subsequently aligned according to the navigation information. Following the alignment, the applicators were inserted via the aiming device and their spatial position verified by control CT imaging.

### Ultrasound:

The GE Logic E10 (General Electric Health Care, Boston, Massachusetts, USA) was used with the curved C1-6 probe or the C2-7 probe, as preferred by the interventionist. Electrodes were placed with needle guide.

### Data collection:

For each control CT scan, the electrode placement was verified in the CAS-One IR software by manually defining the point of the needle tip and at the respective phantom entry location. The resulting verification measurements were used to calculate the interelectrode distances (lateral deviation) and parallelism (angle between the direction vectors) for each electrode pair from a collection of four. For further information see Appendix 1.

### Statistical analyses:

Descriptive statistics were used for presenting interventionists. Boxplots were used to present lateral deviation and parallelism between electrode pairs. For comparing groups with non-normal distribution, the Wilcoxon rank sum test was used, the threshold for statistical significance was set to α < 0.05. For analyses of interindividual differences within the groups a one-way ANOVA was used with a Tukey post-hoc test.

STATA 15 (Stata Corp, College Station, Texas 77,845 USA) was used for the statistical analyses.

### Ethics approval

This article does not contain any studies with human participants performed by any of the authors.

## Results:

### Treatments:

Five radiologists placed four electrodes with ultrasound guidance around five tumours each, in two sets, giving 25 tumours with 100 electrodes and 150 electrode pairs (6 pairs for each tumour). After ten treatments too much air had filled the phantom for a sixth radiologist to perform the treatments.

Six interventionists used CAS-One to place four electrodes around five tumours each, in two sets, giving 30 tumours with 120 electrodes and 180 electrode pairs.

### Lateral distance:

For each set of four electrodes the distances between six electrode-pairs were measured. The optimal distance was 20 mm for four of the pairs, and 28 mm for the diagonal pairs. The absolute values for median deviation from the optimal was 1.3 mm (range 0.0 to 11.3 mm) in the CAS-One group and 7.1 mm (range 0.3 to 18.1 mm) in the ultrasound group.

**Figure 3:**
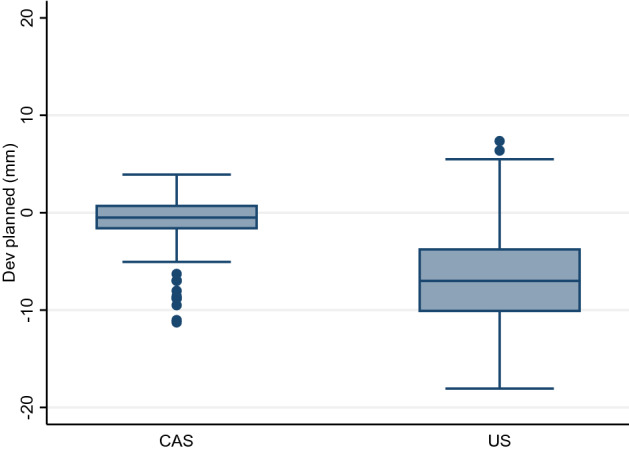
Deviation in millimetre from the optimal distance between the electrode pairs, CAS group and US group.

The deviation from the optimal distance is presented in Fig. 3.

To test the interindividual variance between interventionists a one-sided ANOVA test was performed showing unequal variance. A Tukey’s test for Post-Hoc analysis showed that one of the interventionists using Ultrasound stood out with large differences. A repeated Wilcoxon rank sum test excluding this interventionist still showed a difference between the groups, median of absolute values 1.3 mm (range 0.0 to 11.3 mm) in the CAS group versus 6.7 mm (range 0.3 to 18.1 mm) in the Ultrasound group (p < 0.001).

### Angular error:

The mean angle between electrodes in each pair was 2.7 degrees (95% CI 2.4 to 3.1 degrees) in the CAS-One group and 5.5 degrees (95% CI 5.0 to 6.1 degrees) in the Ultrasound group, the difference was statistically significant (p < 0.001).

**Figure 4:**
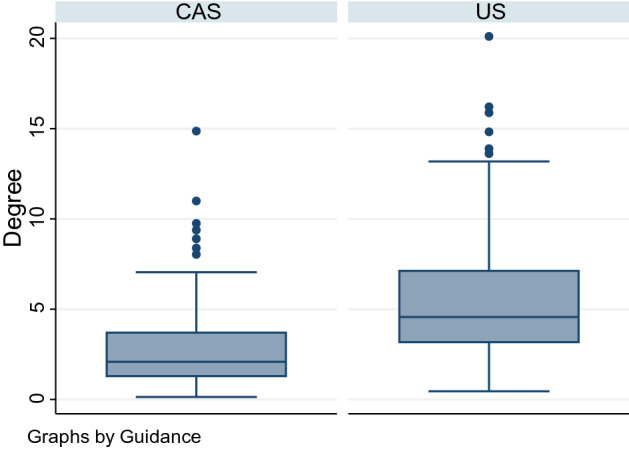
Angle between electrodes in each pair, CAS group and US group.

The angular difference is presented in Fig. 4.

### Time for placing the electrodes:

The mean time per tumour for placing four electrodes was significantly shorter in the Ultrasound group (p < 0.001) with 15:11 min (95% CI 13:05 to 17:18 min) in the CAS group and 6:40 min (95% CI 5:28 to 7:52 min) in the Ultrasound group.

There was no correlation between time and accuracy within the two groups.

## Discussion:

This study shows that stereotactic CT-guided navigation provides higher accuracy both in lateral deviation and parallelism compared with Ultrasound guidance. When considering an active tip of 2 cm, angular deviations may influence the ablation success as no homogeneous ablation volume is generated. As the power settings in the ablation system are typically matched to the electrode spacing solely, the desired electric field strength may not be achieved if the electrodes converge/diverge. This can lead to over-ablation or under-ablation, with the former implying a higher risk of an additional thermal effect or short circuit, and the latter a reversible effect. However, the impact of the parallelism on the oncological outcomes is not widely discussed in current literature and would require further investigation.

The time for placement of the electrodes is faster when using ultrasound than stereotactic navigation; however, the protocol did not allow for needle repositioning, which would have been necessary in certain cases.

Previous publications have shown that the use of a stereotactic CT-based navigation systems in placing applicators for MWA and RFA is safe and accurate^6,7,10–12^. Furthermore, several studies have shown the use of stereotactic navigation in IRE treatment with good results^13–15^.

To our knowledge, there exists no study that compares the accuracy between ultrasound and stereotactic navigation for the placement of IRE applicators.

Sugimoto et al*.* published a review article regarding the role of ultrasonography in IRE-treatment of HCC^9^. They describe different ways to overcome the difficulties in placing multiple parallel electrodes at 1.5–2.0 cm, but they do not present any data regarding accuracy.

The present results are based on electrode placements in a rather rigid phantom model. In real life this would be further complicated by breathing induced liver movement and tissue deformation during the placement of the electrodes^19^. In ultrasound guided ablations there can be a problem with tissue deformation due to the pressure applied by the ultrasound probe. If the liver is compressed by the ultrasound probe during the placement of the applicator, there is a risk of displacement of the electrode when the transducer is removed. This is a minor problem if an intercostal approach is chosen. The problem with electrode displacement is a bigger issue in IRE treatment with multiple electrodes compared to thermal ablations with a single applicator since the transducer-pressure is re-applied several times during the placement of the electrodes. In IRE treatments there is also the issue of space around the electrodes for the ultrasound probe when placing consecutive electrodes where the optimal space for the probe can be blocked by the placed electrodes. In CT-guided electrode placements the pressure deformation is minimal. All this can affect the accuracy in electrode placement in real life and the advantage in accuracy for stereotactic CT-based navigation shown in the phantom could be even greater.

The single centre set up of this study is a limitation of this study. Furthermore, the use of a phantom removes the problems associated with liver motion during clinical use. However, this allows for a direct comparison of the targeting capabilities of the two methods, but it can also be argued that the point of using ultrasound is adaptation to a moving target, where CT guidance relies on methods for controlling breathing related targeting problems with a moving liver, such as tube disconnect or high frequency jet ventilation.

## Conclusion:

The use of stereotactic navigation in placing multiple electrodes for IRE treatment of liver tumours in a phantom model is more accurate than the use of Ultrasound navigation, both in lateral deviation and parallelism of the electrodes.

## Supplementary Information

Supplementary Information.
