# Supplementary material for: Stereotactic navigation versus ultrasound guidance in placing IRE applicators in a liver phantom
Source: Sci Rep. 2021 Oct 26;11:21031. doi: 10.1038/s41598-021-00505-1 (PMC8548523; doi:10.1038/s41598-021-00505-1)
Supplement: Supplementary file 1 — Supplementary Information. [file 41598_2021_505_MOESM1_ESM.docx]

**Appendix 1**

For each IRE configuration, a total of 6 pairs were created (1-2, 1-3, 1-4, 2-3, 2-4, 3-4) that were used for the calculation of the angular and lateral deviation.

The angular deviation was calculated between a pair of electrodes as follows:

$$\theta=\arccos\frac{\overline{{Entry}_{0}{Tip}_{0}} \cdot\overline{{Entry}_{1}{Tip}_{1}}}{\left| \overline{{Entry}_{0}{Tip}_{0}} \right|\cdot\left| \overline{{Entry}_{1}{Tip}_{1}} \right|} ,$$

where $\overline{{Entry}_{0}{Tip}_{0}}$ and $\overline{{Entry}_{1}{Tip}_{1}}$ denote the directing vectors from the 3D entry and tip coordinates of the respective needle.

The lateral deviation was calculated as the closest distance from the tip of one needle to the line through the entry and tip of the second needle:

$$d= \frac{\left| \overline{{Tip}_{1}{Tip}_{0}}\times\overline{{Entry}_{0}{Tip}_{0}} \right|}{\left| \overline{{Entry}_{0}{Tip}_{0}} \right|} .$$
